# Simulation Approach for Random Diffusion of Chloride in Concrete under Sustained Load with Cellular Automata

**DOI:** 10.3390/ma15134384

**Published:** 2022-06-21

**Authors:** Junjun Ma, Pengzhen Lin

**Affiliations:** Key Laboratory of Road and Bridge and Underground Engineering of Gansu Province, Lanzhou Jiaotong Unversity, Lanzhou 730070, China; majjlz@163.com

**Keywords:** chloride ion, concrete, cellular automata, diffusion, model validation

## Abstract

Steel bar corrosion caused by chloride is the major reason for concrete structure durability failures in a corrosive environment. An accurate simulation of chloride ion diffusion in concrete is hence critical to durability design, maintenance, and reinforcement of concretes in erosive environments. To accurately simulate actual chloride ion diffusion in concretes, an improved three-dimensional neighborhood type is proposed according to the mechanism of chloride ion diffusion in concrete, and a three-dimensional cellular automaton model (3D CA model) for describing the diffusion process of chloride in concrete is established based on this neighborhood type. The accuracy and correctness of simulation results obtained from the 3D CA model were verified by comparison with Fick’s second law analytical solutions. Based on the 3D CA model, an improved modified 3D CA model is developed (3D RTCA model) which takes into account random chloride ion distribution in concrete, the time dependence of the coefficient of chloride ion diffusion, and the structure stress level effect on chloride ion diffusion. Numerical simulation results reveal that the 3D RTCA model has higher calculation accuracy in predicting long-term concentration of chloride in concretes, and the simulation results are closer to experimental findings than analytical results obtained based on Fick’s second law. Compared with Fick’s second law analytical solutions, the 3D RTCA model can reflect more truly the cross-sectional stress level effect on chloride ion diffusion through simple local evolution rules. Besides, the 3D RTCA model can genuinely describe the randomness and uncertainty of the chloride diffusion process. The 3D RTCA model developed in the current study provides a novel perspective and method to investigate chloride ion diffusion in concrete from structural level.

## 1. Introduction

Concrete structure durability, involving structural safety, applicability, and durability, has become the focus of research in the world of engineering [1]. Steel bar corrosion due to chloride ion corrosion is the most important reason for durability failure of concrete structures in corrosive marine environments in the long term [2,3]. Hence, the accurate simulation of these erosive materials, such as chloride ions, is essential for durability design, maintenance, and reinforcement of concrete structures in corrosive marine environments.

Recently, great progress has been made in the simulation of aggressive substances such as chloride ions, and novel approaches and models to simulate chloride ion diffusion in concretes have been proposed. Collepardi [4] introduced chloride ion diffusion in concretes in marine environments using Fick’s second law and Mass Conservation Law. Subsequently, Fick’s second law was extensively applied by other researchers to simulate the chloride diffusion process inside concrete structures. Mangat [5] and Zhang [6] deduced a differential equation for chloride ion diffusion in concrete by introducing time-dependent parameters into the diffusion coefficient to take into account time effect on chloride ion diffusion. A mathematical model describing chloride ion permeation in saturated concrete was established by Xi [7] which takes into account the influences of material water–cement ratio, environmental temperature, cement type, and curing time on the chloride ion diffusion effect [7,8]. Suryavanshi [9] proposed an approach to evaluate chloride diffusion coefficient in concrete through chloride ion erosion test results on reinforced concrete slabs. In addition to the above model, some service life models, such as the mathematical statistics method [10], meshless Galerkin method [11,12], finite element method [13,14], artificial neural network model [15,16], and Novel Machine Learning Techniques [17] have been used for the prediction of the distribution of chloride concentration in concrete. Simultaneously, to facilitate the description of the uncertainty and randomness of the chloride diffusion process in concrete, some scholars have proposed a series of probabilistic prediction models for describing the chloride diffusion process in concretes by taking the randomness of the main calculation parameters into consideration [18,19,20]. In addition, considering the stringent requirements for boundary conditions when solving based on Fick’s second law, some scholars have proposed that Fick’s second law is solved by a new numerical algorithm called cellular automata, which could be well implemented for the solution of chloride diffusion problems with complex boundary conditions.

Cellular automata (CA), also known as cell automata, or lattice automata, is a discrete grid dynamic model in space and time with the capability of simulating the spatiotemporal evolution of complex systems [21]. Since it was first introduced, cellular automata models have been extensively applied in various fields of scientific, military, economic, and social research [22,23,24]. In recent years, CA models have been applied and developed in the field of civil engineering. Biondini used cellular automata for the analysis of frame structure and concrete beam durability under external chloride ion diffusion erosion, and evaluated their service life [21]. Podroužek [25] applied the cellular automata model to analyze the evaluation of structural performance degradation of bridge structures under chloride erosion. Considering the effects of erosion time and depth on chloride diffusion coefficient, a cellular automaton model was proposed by Cao [26]. Titi [27] found that the two-dimensional cellular automata model has higher accuracy in the durability evaluation of concrete structures compared with one-dimensional cellular automata models. In addition, there has been consideration of the influences of water transport, aggregate distribution, ion absorption, and chloride ion diffusion. Literature [28] considered the effect of the concentration of chloride ions on reinforcement erosion, and established an analysis method for evaluating the durability and reliability of reinforced concrete structures under chloride ion corrosion by using 2D CA technology. Considering the migration phenomenon and corrosion current of oxygen and chloride ions, German et al. [29] analyzed the relationship between corrosion time and chloride ion diffusion coefficient, chloride ion threshold, and thickness of the protective layer of steel reinforcement by combining CA and other numerical methods. Based on mesoscopic features, Ruan [30] proposed a refined cellular automaton approach that improves the efficiency of simulation of chloride ion erosion processes in concrete. The above research using cellular automata to simulate the chloride diffusion process in concretes is limited to a two-dimensional (2D) plane, and cannot simulate the chloride ion three-dimensional erosion process in concrete. However, in an erosive environment, three-dimensional (3D) erosion often exists in the location where the concrete structure contacts the erosive environment. In addition, in the actual chloride ion diffusion, chloride ion diffusion coefficient is a time-dependent variable, not a constant value, because continuous development of cement hydration reduces the original pore distribution of the structure, which delays chloride ion migration in concretes. Along with erosion time effect on chloride ion diffusion coefficient, the load applied on the structures also affects chloride ion diffusion coefficient. Many researchers have not only conducted a large number of experimental research works on chloride ion diffusion in stressed concretes, but also have established prediction models for chloride ion diffusion in concrete under various load conditions [31,32]. However, the models only consider the stress level effect at the most unfavorable position of the cross-section on chloride ion diffusion coefficient. Regarding the stress level effect on chloride ion diffusion coefficient as a fixed value, it does not take the change in structural stress level along with the height and length of the structure into consideration. Therefore, the variation of diffusion coefficient with erosion time and structural stress level should also be considered in simulating chloride ion diffusion in concretes based on the CA model.

Therefore, considering the random effects, time-varying characteristics, and stress level changes (Size and Direction) of chloride ions in concrete on the diffusion of chloride ions, a 3D CA model for diffusion of chloride ions into concrete is established in this paper. First, according to the random walk phenomenon of chloride ion in concretes, local evolution rules describing chloride ion diffusion into concrete are given from based on Probability Theory. On this basis, a 3D CA model for the simulation of chloride ion diffusion in concretes was proposed. Then, the developed 3D CA model was validated using Fick’s second law. Secondly, taking further consideration of the influence of erosion time and structural stress level on chloride ion diffusion and randomness of chloride ion diffusion, an improved cellular automata model, called the 3D RTCA model, was proposed. Finally, the accuracy of the developed model was verified by experimental data, and compared with Fick’s second law analytical solutions, indicating model effectiveness and its ability to describe material variability.

## 2. The 3D CA Model to Simulate Chloride Ion Diffusion Process in Concretes

### 2.1. Model of Chloride Diffusion in Concrete

Concrete erosion due to chloride ions is mainly diffusion, and Fick’s second law can usually describe its transmission process. At a constant diffusion coefficient without considering the change with time and position, the second-order partial differential equation of Fick’s second law are further discussed as the following linear equation [21,33]:

(1) $$D\nabla^{2}C=\partial C/\partial t$$

where *C* = *C*(*x*, *y*, *z*, *t*) is the concentration of chloride ions evaluated at point (*x*, *y*, *z*) and time *t*; *D* is the diffusion coefficient of chloride ions; ∇^2^ = ∇·∇, where ∇*C* = grad *C*. For semi-infinite concrete eroded by chloride ions, a solution for the above equation is [6,34]:

(2) $$C\left(x,y,z,t\right)=C_{0}+C_{s}-C_{0}\left[1-\prod_{d=1}^{3}\mathrm{erf}\left(0.5h_{d}D^{0.5}t^{0.5}\right)\right]$$

where *C*_0_ and *C_s_* are initial and surface chloride ion concentrations in concrete, respectively, *h_d_* is distance from evaluation point (*x*, *y*, *z*) to the concrete surface, and erf is the error function. This analysis method could be applied to simulate the concentration of chloride ions in concretes, but it can only solve the chloride ion diffusion problem with classic simple boundaries, such as orthogonal boundaries. Therefore, other solutions are usually required for the chloride diffusion equations under complex boundary conditions. In the paper, the analytical solutions of diffusion equations are obtained through a unique numerical calculation method called cellular automata.

### 2.2. Overview of CA

**Figure 1:**
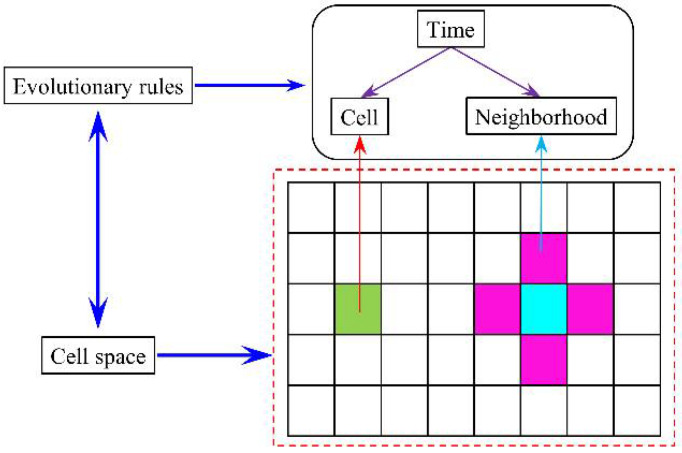
Relationship between components of cellular automata.

CA are dynamic grid systems that define the research object as a cell space of finite and discrete states and evolve in discrete time dimensions based on certain local evolution rules [22,23]. Cellular automata, unlike general dynamic models, do not rely on strictly defined physical functions and equations but are made up of rules obtained by a series of models. All models that satisfy the mentioned rules could be considered as cellular automata models. The cellular automata are mainly composed of cellular, cellular space, cellular domain, local evolution rules, cellular state, boundary conditions, and initial conditions [21]. Their interrelationship is shown in Figure 1, with the cellular state representing chloride ion concentration at that location. CA-simulated chloride ion diffusion through local evolution rules: the state *C*(***s****_i_*, *t* + ∆*t*) at each cell ***s****_i_* at time *t* + Δ*t* is updated synchronously based on the state *C*(***s****_i_*, *t*) at cell ***s****_i_* and *C*(***s****_n_*, *t*) at their neighborhood cell ***s****_n_* at the time *t*. Thus, the local evolution rules of cellular automata can be expressed as [21,30]:

(3) $$C\left(s_{i},t+\Delta t\right)=f\left(C\left(s_{0},t\right),C\left(s_{1},t\right)\cdot \cdot \cdot C\left(s_{i},t\right)\cdot \cdot \cdot C\left(s_{n},t\right)\right)$$

where the function *f*(·) is the local evolution rule of cellular automata and *C*(***s****_i_*, *t* + ∆*t*) is the state (concentration of chloride ions) of cell ***s****_i_* at time *t* + Δ*t*.

### 2.3. Evolutionary Rules

**Figure 2:**
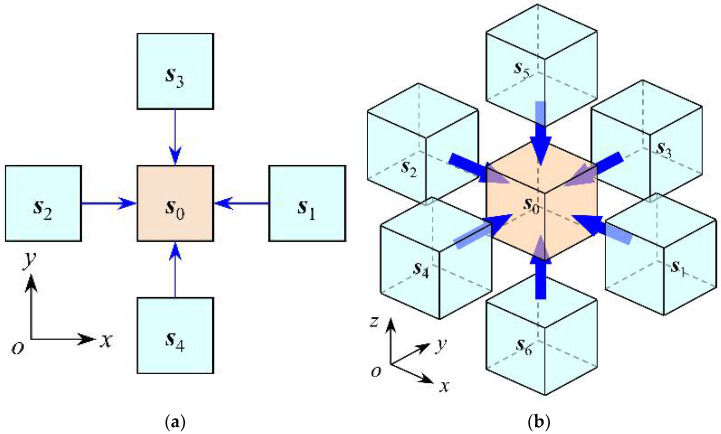
Type of neighborhood for 2D and 3D cellular automata. (**a**) Two-dimensional; (**b**) Three-dimensional.

According to the principle of CA, when using CA to solve the problem of chloride ion diffusion, it is necessary to first determine the type of cell neighborhood, that is, to determine which neighboring cells around the cell have an impact on the intracellular chloride ion diffusion effect [21,27]. Figure 2a shows the von Neumann neighborhoods for solving the problem of planar chloride ion diffusion. Since only four neighborhood cells of ***s***_1_, ***s***_2_, ***s***_3_, and ***s***_4_ are used, this type of neighborhood can only simulate the diffusion process of chloride ions in the plane, and cannot simulate the diffusion process of chloride ions in the overall structure. Therefore, it is necessary to propose a neighborhood type that can simulate the three-dimensional chloride ion diffusion process. Taking the central cell ***s***_0_ shown in Figure 2b as an example, it is affected by the diffusion of chloride ions in the directions of ***s***_1_, ***s***_2_, ***s***_3_, ***s***_4_, ***s***_5_, and ***s***_6_. If the neighbor cells in these six directions are taken as the neighborhood cells of the central cell, a type of neighborhood that simulates the 3D chloride diffusion process can be established. The improved relationship between central cell ***s***_0_ and neighbor cell ***s****_k_* can be described as:

(4) $$s_{k}=s_{0}+L_{k}e_{k},k=1,2,3,4,5,6$$

where, ***e****_k_* is the unit vector where the central cell ***s***_0_ points in the direction of adjacent cell ***s****_k_*; *L_k_* is the distance between the center cell ***s***_0_ and the neighboring cell ***s****_k_*.

According to the above proposed 3D cellular neighborhood types and evolutionary rules (3), the diffusion process of chloride ions in concrete can be described by Equation (5):

(5) $$C\left(s_{0},t+\Delta t\right)=\alpha_{0}C\left(s_{0},t\right)+\sum_{k=1}^{6}\beta_{k}C\left(s_{k},t\right)$$

where *C*(***s***_0_, *t*) and *C*(***s***_0_, *t* + ∆*t*) are the chloride concentrations represented by the central cell ***s***_0_ at the time of *t* and *t* + ∆*t*, respectively; *C*(***s****_k_*, *t*) is the chloride concentration represented by neighboring cell ***s****_k_* in the *k* direction of the central cell ***s***_0_ at the time of *t*; *α*_0_ is the influence coefficient of the central cell ***s***_0_ at time *t* on the central cell ***s***_0_ at time *t* + ∆*t*; and *β_k_* is the influence coefficient of the neighbor cell ***s****_k_* at time *t* on the central cell ***s***_0_ at time *t* + ∆*t*. According to the law of conservation of mass, the following relationship is satisfied between the coefficients of influence:

(6) $$\alpha_{0}+\sum_{k=1}^{6}\beta_{k}=1$$

Equation (5) is the chemical kinetic equation for solving the diffusion process of chloride ions in concrete using CA. To regulate the above evolution according to the given chloride diffusion coefficient, the relationship between the chloride ion diffusion rate and the influence coefficient is established with the law of conservation of mass, and the specific process is as follows. First, taking the central cell ***s***_0_ shown in Figure 2b as an example, according to the law of conservation of mass, the following equation is satisfied between the chloride ion content in cell ***s***_0_:

(7) $$M\left(s_{0},t+\Delta t\right)=M\left(s_{0},t\right)+M_{in,k}-M_{out,k}$$

where ∆*t* is the time step; *M*(***s***_0_, *t* + ∆*t*) and *M*(***s***_0_,*t*) are the chloride ion content in cell ***s***_0_ at the moment of *t* + ∆*t* and *t*, respectively; and M_*in*,*k*_ and M_*out*,*k*_ are the chloride ion content inflow and outflow of cellular ***s***_0_ in the *k* direction, respectively. According to the definition of diffusion flux, the relationship between M_*in*,*k*_ and M_*out*,*k*_ and diffusion flux can be expressed as:

(8) $$M_{in,k}=\Delta tA_{k}J_{i,k}\left(s_{0},t\right),M_{out,k}=\Delta tA_{k}J_{o,k}\left(s_{0},t\right)$$

where *A_k_* is the area through which cell ***s***_0_ diffuses along the *k* direction, and *J_o_*_,*k*_ and *J_i_*_,*k*_ are the diffusion fluxes of chloride ions outflowing and inflowing in the *k* direction of cell ***s***_0_ from *t* to *t* + Δ*t*, respectively.

Combining Equations (7) and (8), the chloride content in cellular ***s***_0_ can be simplified to

(9) $$M\left(s_{0},t+\Delta t\right)=M\left(s_{0},t\right)+\Delta t\sum_{k=1}^{6}\left[A_{k}\left(J_{i,k}\left(s_{0},t\right)-J_{o,k}\left(s_{0},t\right)\right)\right]$$

Furthermore, according to Fick’s first law, the diffusion fluxes of chloride ions in each direction can be obtained from the concentration of chloride ions in neighbor cells in that direction, as shown in Equation (10).

(10) $$J_{o,k}\left(s_{0},t\right)=-J_{i,k}\left(s_{0},t\right)=D\left[C\left(s_{0},t\right)-C\left(s_{k},t\right)\right]/L_{k}$$

Substituting Equation (10) into (9), the improved relationship is presented as follows.

(11) $$M\left(s_{0},t+\Delta t\right)=M\left(s_{0},t\right)-D\Delta t\sum_{k=1}^{6}\left\{L_{k}\left[C\left(s_{0},t\right)-C\left(s_{k},t\right)\right]\right\}$$

According to the law of large numbers, the relationship between chloride ion content and concentration in cell ***s***_0_ is presented as Equation (12).

(12) $$M\left(s_{0},t\right)=C\left(s_{0},t\right)\cdot L_{k}^{3}$$

Combining Equations (11) and (12), the evolution equation of concentration of chloride ions in cell ***s***_0_ is presented as Equation (13).

(13) $$C\left(s_{0},t+\Delta t\right)=C\left(s_{0},t\right)-D\Delta t\sum_{k=1}^{6}\left\{\frac{1}{L_{k}^{2}}\left[C\left(s_{0},t\right)-C\left(s_{k},t\right)\right]\right\}$$

In the case of cube cells (*L_k_* = *d*), comparing Equations (5) and (13), the relationship between the coefficient of evolution and the coefficient of diffusion is presented as Equation (14):

(14) $$\alpha_{0}=1-\sum_{k=1}^{6}\beta_{k},\beta_{k}=\beta_{1}=D\Delta t/\delta^{2}$$

where *d* is the cell size.

As a result, combined with Equations (5) and (14), a 3D CA model was established to simulate the diffusion process of chloride ions in concrete. However, other corrosive effects of chloride ions in concrete, such as penetration, need to be reestablished in combination with other theories.

It is observed from Equation (14) that diffusion coefficient *D* is proportional to the coefficient of evolution α_0_ or *β*_1_ and the time step, and inversely proportional to the cell size. Some literature has proved that the values *α*_0_ = 1/2 and *β*_1_ = 1/12 usually led to high accuracy of the calculation results of CA [21,30]. However, in the actual chloride ion diffusion process, chloride ion diffusion coefficient *D* is not a fixed value, and varies with other factors such as external environment and temperature and other factors. Therefore, to ensure the accuracy of the calculation, the cell size and time step should be reasonably selected before modeling and analysis to achieve balance between the two parameters.

### 2.4. Calculation Process and Steps for the 3D CA Model

**Figure 3:**
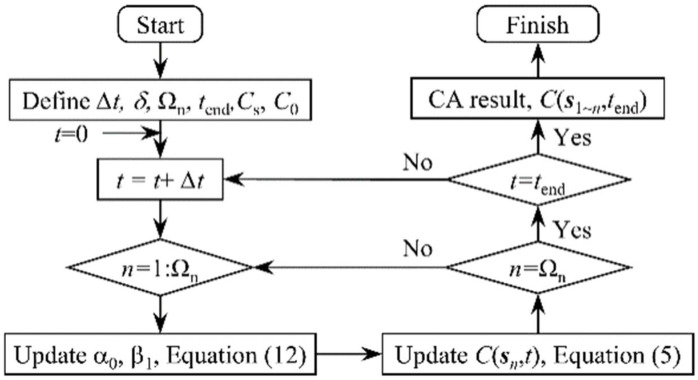
Calculation process and steps for the 3D CA model.

The simulation of the chloride diffusion process in concrete can be conducted based on the proposed CA model. Since the coefficient of evolution *α*_0_ or *β*_1_ in the CA model is dynamically updated with parameters such as diffusion coefficient of chloride ions and time step, the specific solution details of the model, such as time step and solution sequence, should be specified before solving. The specific solution process is illustrated in Figure 3. In this manuscript, all CA models involved are constructed and solved with MATLAB software (R2021b, LanZhou, China). The steps to solve the model are as follows:

(1) The initial calculation parameters of the model are set at first, including the time step, the total time, and the cell size. Next, the study objects are meshed according to the cell size. Then, a set of state variables for recording chloride ion concentrations is established based on the number of units after division. Finally, the state variable of each cell set an initial value based on its location, including the boundary concentration and initial concentration.
(2) At each time step, the evolution coefficient of the cell is updated according to the current time step and the chloride ion diffusion coefficient in each cell, and on this basis, the state variable of each cell in the time step is updated according to the evolution rules. For the whole solving process of the model, the evolutionary coefficients and state variables of the cell are continuously updated until all cells and the entire time step are covered.
(3) Obtain the final concentration of each cell and the concentration distribution of chloride ions throughout the concrete cross-section.

### 2.5. Validation of the 3D CA Model

**Figure 4:**
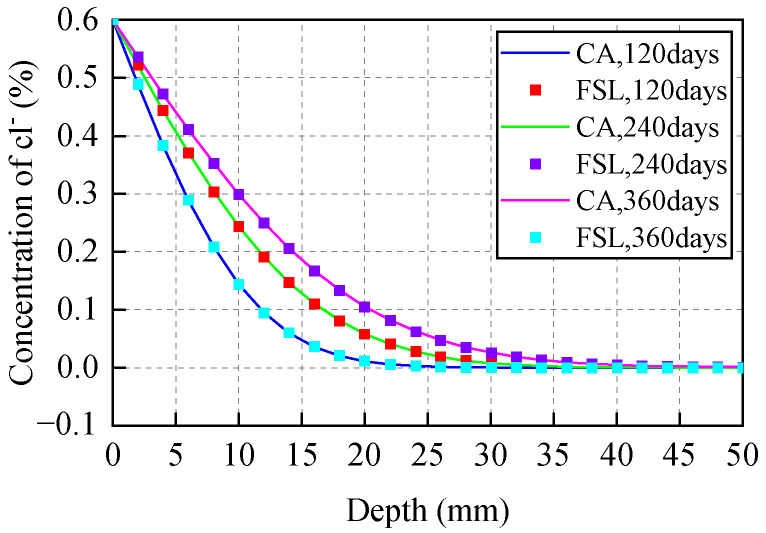
Chloride ion concentration distribution in concrete determined by CA and FSL methods.

**Figure 5:**
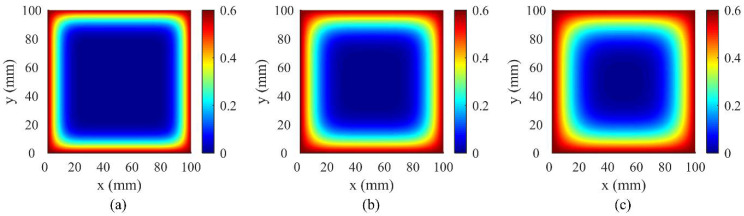
Simulation results for chloride ion concentration in concrete cross-section under various corrosion times: (**a**) *t* = 120 days; (**b**) *t* = 240 days; (**c**) *t* = 360 days.

Through the comparison of simulation results of the 3D CA model with Fick’s second law analytical results, model accuracy was verified. Assuming a concrete specimen with cross-section 100 mm × 100 mm and height 400 mm, initial chloride ion concentration on the concrete specimen surface was set as *C*_0_ = 0.6%. The diffusion coefficient was adjusted at *D* = 3.5 × 10^−12^ m^2^/s. The grid size was set as *d* = 0.1 mm. The erosion probability was set as *α*_0_ = 0.5. Then, under the condition that the above parameters are known, the evolution time step ∇*t* = 0.0041 days can be obtained according to Equation (14). Figure 4 shows the concentration distribution of chloride ions in a concrete cross-section predicted by the 3D CA model and Fick’s second law (FSL) under different corrosion time. Figure 5 presents the simulation results of chloride ion concentration in concrete cross-section at various corrosion times.

From Figure 4, chloride ion concentration in concrete cross-section increases with time and decreases with depth. A good correlation is witnessed between analysis results of FSL and those predicted by the CA model, and the maximum deviation between the two calculations does not exceed 3%. This demonstrates that the 3D CA model proposed previously is feasible and reasonable to simulate the chloride ion diffusion process in concretes.

## 3. Improved 3D CA Model

### 3.1. Stress Level Effect on Chloride Ion Diffusion

For concrete structures in actual engineering, most of the structures are subjected to loads during service. However, before the load was applied, the concrete had many small cracks, pores, and micro-defects inside due to the characteristics of the structure itself. Therefore, once a load acts on the structure, stress concentration will occur in these places, resulting in a significant variation in concrete internal pore structure, thereby affecting the erosion ability of chloride ions in concrete [31,35]. Now that a large number of studies investigate the influence of applied load on chloride ion diffusion, load effect on chloride ion diffusion is generally taken into account by the multiplication of the original chloride ion diffusion coefficient by the corresponding influence coefficient [36,37]. Therefore, under bending loads, the relationship between concrete cross-section stress distribution and chloride ion diffusion coefficient in concretes is stated as:

(15) $$D_{s}=K_{s}\cdot D_{0}$$

where *D*_0_ and *D_s_* are chloride ion diffusion coefficient in concretes under no stress and after considering load, respectively, and *K_s_* is load influence coefficient on chloride diffusion coefficient. In this paper, the expression of the stress influence coefficient used is as follows [38]:

(16) $$K_{s}^{t}=1.0+0.9598\eta_{t}-0.3608\eta_{t}^{2}$$

(17) $$K_{s}^{c}=1.0-1.6626\eta_{c}+2.2560\eta_{c}^{2}$$

where *η_c_* is compressive stress level, *η_c_ = σ/f_c_*; *η_t_* is the tensile stress level, *η_t_ _=_ σ/f_t_*; *f_t_* is the design value of concrete tensile strength; and *f_c_* is the design value of concrete compressive strength.

### 3.2. Time-Varying Characteristics of Chloride Ion Diffusion

The 3D CA model proposed previously can well simulate and analyze the basic chloride ion diffusion in concretes. On the other hand, in actual structure, due to the continuous progress of the late hydration of concrete, the original pores, and micro-cracks, etc., are constantly changing, which leads to changes in the permeability of the concrete structure [39,40]. In addition, chloride ion diffusion coefficient in concrete, as a key index reflecting concrete structure permeability, should also vary with concrete curing time. Therefore, it will greatly improve the accuracy of the model by introducing time effect on diffusion coefficient in cellular automaton model. The variation law of chloride ion diffusion coefficient in concretes with corrosion time is well presented by Equation (16) [41,42,43]:

(18) $$D_{t}=D_{0}{\left(t_{0}/t\right)}^{m}$$

where *m* is the time-dependent constant of chloride ion diffusion coefficient in concrete, and *D_t_*, and *D*_0_ are the chloride diffusion coefficients in concrete at time *t*_0_ and *t*, respectively. Generally, the reference period *t*_0_ is the 28th day after the concrete is premixed.

### 3.3. Stochastic Effect of Chloride Ion Diffusion Process

The diffusion of chloride ion process in concrete often shows stochastic effects [44,45]. Considering the randomness and uncertainty of chloride ion diffusion, this manuscript has improved the 3D CA model proposed above. In the erosion, the influence coefficient (α_0_ and *β_k_*) used to determine the cell evolution rule will not be a fixed number but a new variable (*j*_0_ and *l_k_*) determined by the random probability density distribution *f*(*y*). For this reason, the relationship between the influence coefficients before and after the improvement can be simply expressed by Equation (19).

(19) $$\varphi_{0}=\left(1-\psi_{0}\right)\alpha_{0},\lambda_{k}=\left(1-\psi_{k}\right)\beta_{k},k=1,2,3,4,5,6$$

In the case of ensuring that the original influence coefficient is unchanged, based on the law of conservation of mass, the new random variable must satisfy the following relationship:

(20) $$\frac{1}{2}\psi_{0}+\frac{1}{12}\sum_{k=1}^{6}\psi_{k}=0$$

**Figure 6:**
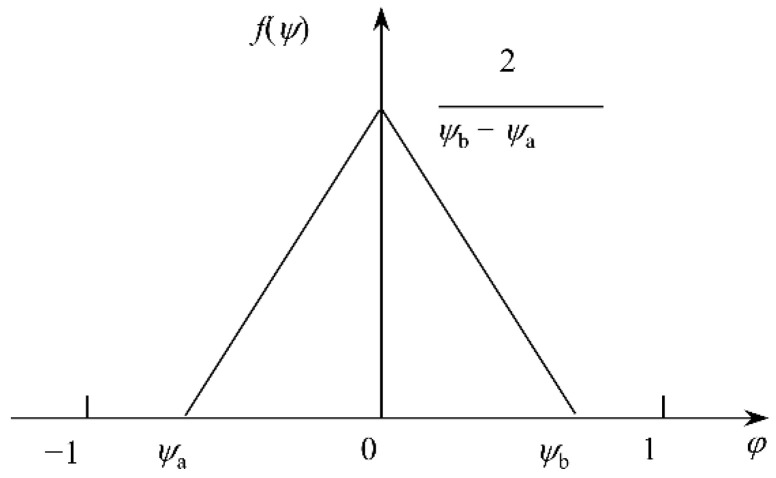
The probability density function of the triangular distribution.

From Equation (20) and the value range of the random variable *y*, it is observed that the probability density distribution function *f*(*y*) used to determine the random variable *y* must adopt the symmetric function distributed within the interval (−1:1) To satisfy the above distribution characteristics, a triangular distribution is used in this paper [21], and its probability density distribution function is shown in Figure 6.

### 3.4. A 3D RTCA Model to Simulate the Chloride Ion Diffusion Process in Concretes

Considering random-effect and time-varying characteristics of chloride ion diffusion in concrete, and the effect of stress level on chloride ion diffusion, the relationship between the improved influence coefficients and erosion time can be described by Equation (19).

(21) $$\varphi_{0}=\left(1-\psi_{0}\right)\left(1-6K_{s}D_{0}\Delta t{\left(t_{0}/t\right)}^{m}\delta^{-2}\right),\lambda_{k}=\left(1-\psi_{k}\right)K_{s}D_{0}\Delta t{\left(t_{0}/t\right)}^{m}\delta^{-2},k=1,2,3,4,5,6$$

The evolution equation in the simulation of the chloride ion diffusion process in concrete by the CA method can be expressed as:

(22) $$C\left(s_{0},t+\Delta t\right)=\varphi_{0}C\left(s_{0},t\right)+\sum_{k=1}^{6}\lambda_{k}C\left(s_{k},t\right)$$

An improved 3D CA model, called the 3D RTCA model, can be obtained by combining Equations (20)–(22). Compared with the CA model, the RTCA model can not only consider the time-varying characteristics of the chloride ion diffusion process under load, but also reflect the randomness and uncertainty of the diffusion process of chloride ions, which can better reflect the engineering reality.

**Figure 7:**
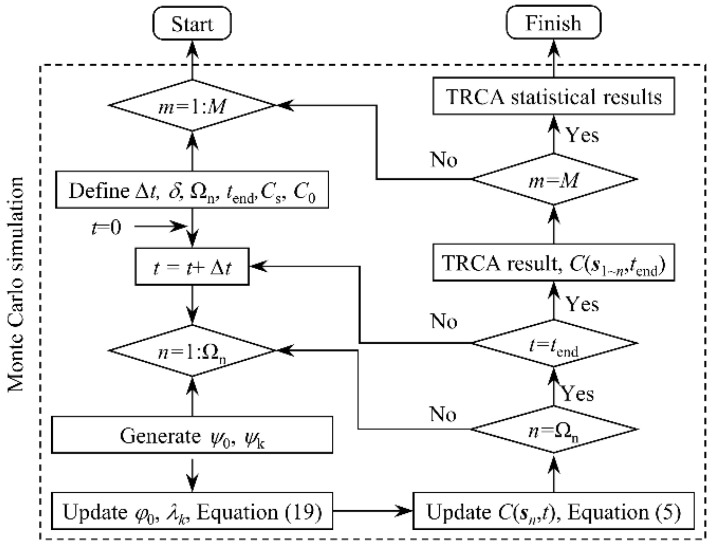
Calculation process and steps for the 3D RTCA model.

Based on the above improved cellular automaton model, the stochastic diffusion process of chloride ions in concrete can be simulated using the Monte Carlo simulation method. The solution steps of the RTCA model shown in Figure 7 are basically the same as that of the CA model, but slightly different, which is mainly reflected in the following two aspects: (1) In the new calculation steps, the influence coefficients in step 2 are not a constant, but a set of random variables related to factors such as erosion time, cross-section stress, etc.; (2) Monte Carlo simulation was added to the RTCA model calculation step.

## 4. Experimental Validations

### 4.1. Chloride Ion Diffusion in Unstressed Concretes

**Figure 8:**
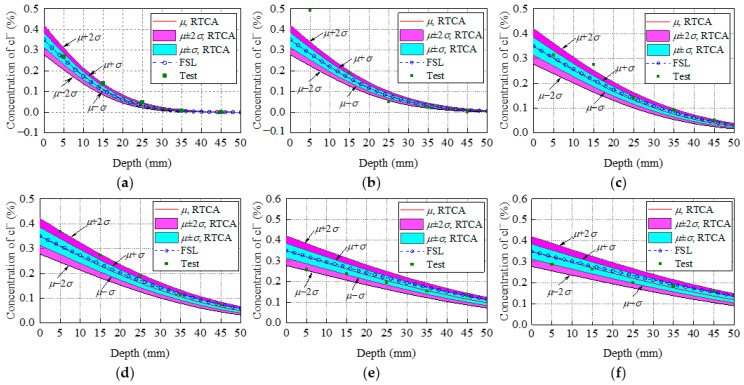
Comparisons between the statistical result (*μ σ*) using the 3D RTCA model, FSL analytical solution, and test data for PC concrete: (**a**) *t* = 0.5 year; (**b**) *t* = 1 year; (**c**) *t* = 2 years; (**d**) *t* = 3 years; (**e**) *t* = 6 years; (**f**) *t* = 8 years.

**Figure 9:**
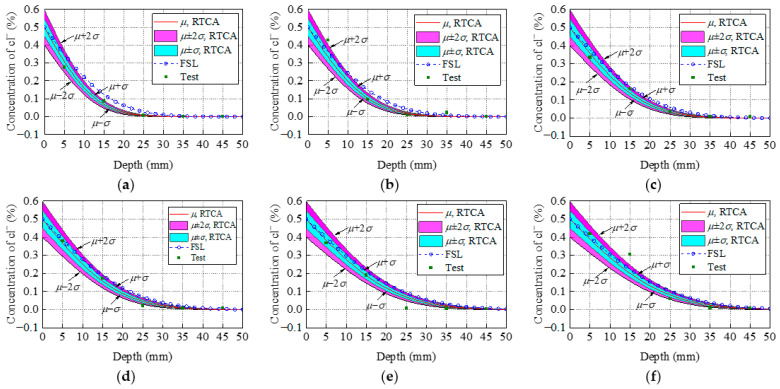
Comparisons between the statistical result *μ, σ* using the 3D RTCA model, FSL analytical solution, and test data for fly ash concrete: (**a**) *t* = 0.5 year; (**b**) *t* = 1 year (**c**) *t* = 2 years; (**d**) *t* = 3 years; (**e**) *t* = 6 years; (**f**) *t* = 8 years.

To investigate the accuracy of the developed 3D RTCA model, the results obtained from the simulation model are compared with experimental results in Thomas and Bamforth [46]. The concrete specimen size is 100 mm × 100 mm × 1000 mm. The corresponding CA simulation for the above experiment process was conducted. According to the experimental results [46] and research from conferences [44,45], the statistical input parameters and the distribution type that best simulates the experimental results are listed in Table 1. In the 3D RTCA model, the size of the cell and the erosion probability during the simulation process were defined as *d* = 0.1 mm, *α*_0_ = 0.5, and *β*_1_ = 1/12, respectively. The stress influence coefficient was defined as *K_s_* = 1. The random variable *y* was calculated according to the triangular distribution function. The time step varies with time and random variable *y*, and its value was defined according to Equations (14) and (19). According to the above parameters, chloride ion concentration distribution in a concrete cross-section after 0.5, 1, 2, 3, 6, and 8 years was randomly predicted. With reference to a sample of 1000 simulations, comparisons between the statistical result (average value *μ*, standard deviation *σ)* using the 3D RTCA model, Fick’s second law (FSL) analytical solution, and the test value for the two concrete mixes are shown in Figure 8 and Figure 9, where the red curve indicates the statistical result, the blue dotted line indicates the analytical solution, and the purple square indicates the experimental data. At the same time, to describe the degree of dispersion of the experimental results, Figure 8 and Figure 9 also show the average chloride ion concentration deviation *σ* and 2*σ* at various locations of the concrete section.

**Figure 10:**
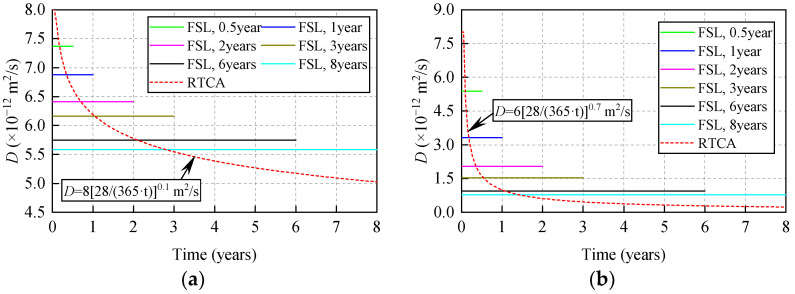
Variations of the diffusion coefficient with corrosion time in the two calculation methods: (**a**) PC concrete; (**b**) fly ash concrete.

From the statistical result of PC concrete shown in Figure 8, it can be seen that all experimental data points, except for a few experimental data points, are in the interval (*μ* – 2*σ: μ* + 2*σ*) and more than half of these experimental data points are in the interval (*μ* – *σ: μ + σ*). Meanwhile, the same conclusions drawn for Figure 8 apply to the fly ash concrete shown in Figure 9. In summary, this indicates that the 3D RTCA model can better describe the uncertainty and randomness of chloride ion diffusion in concrete, and reflect actual chloride ion distribution in concrete cross-section. In addition, it also can be observed from Figure 8 and Figure 9 that the statistical results (average value *μ* of chloride ion concentration in cross-section obtained by applying the 3D RTCA model are greater than those obtained by FSL analytical solution, especially for fly ash concrete, and the predicted values of the 3D RTCA model are closer to the test values for two types of concrete. To explain, Figure 10 presents the variations of the diffusion coefficient of chloride ions with corrosion time in both calculation methods, where the solid and dashed lines represent the chloride diffusion coefficients used by the FSL and 3D RTCA models, respectively.

As shown in Figure 10, when predicting the concentration of chloride ions in concrete using the FSL method, while the influence of time on the diffusion coefficient of chloride ions can be reflected to a certain extent, the change in chloride ion diffusion coefficient with time cannot be considered in the time step. The diffusion coefficient is considered to be constant during this application. Conversely, when predicting the concentration of chloride ions in concrete using the 3D RTCA model, the chloride ion diffusion coefficient is updated in real-time with the time step. As a result, compared with FSL, the 3D RTCA model can truly reflect the time-varying characteristics of the chloride ion diffusion coefficient when predicting chloride ion concentration in concretes. The 3D RTCA model has higher accuracy in predicting chloride ion concentration in concretes.

**Figure 11:**
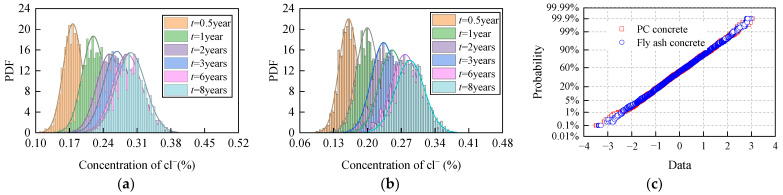
Probability mass functions and corresponding normal probability density distribution models for chloride ion concentration at 10 mm from concrete surface under different corrosion time: (**a**) PC concrete; (**b**) fly ash concrete; (**c**) the test analysis results after standardization.

Figure 11 presents the functions of probability mass and corresponding normal probability density distribution models of chloride ion concentration at 10 mm from the concrete surface under different corrosion times for a sample of 1000 Monte Carlo realizations. Figure 11a,b show the statistical results of PC concrete and fly ash concrete, respectively, where the bar graph represents the probability mass function, and the line represents the probability density distribution function.

It is observed that chloride ion concentration PDF at different corrosion times is bell-shaped, and that becomes flatter and wider as the erosion time increases. The significance test values obtained by the K–S test are greater than 0.05, indicating that the concentration of chloride ions in the concrete follows the normal distribution. Furthermore, the test analysis results after the standard normalization of concentration of chloride ions also show that the concentration of chloride ions follows the normal distribution, and the test results are shown in Figure 11c.

**Figure 12:**
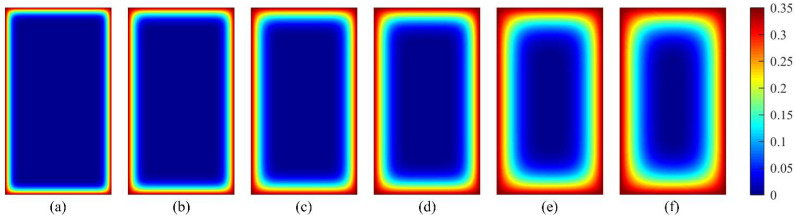
Simulation results (average value *μ*) of the concentration of chloride ions in cross-section of PC concrete under different corrosion times: (**a**) *t* = 0.5 year; (**b**) *t* = 1 year; (**c**) *t* = 2 years; (**d**) *t* = 3 years; (**e**) *t* = 6 years; (**f**) *t* = 8 years.

**Figure 13:**
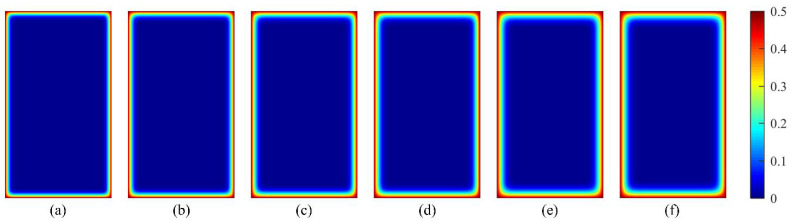
Simulation results (average value *μ*) of chloride ion concentration in cross-section of fly ash concrete under different corrosion times: (**a**) *t* = 0.5 year; (**b**) *t* = 1 year; (**c**) *t* = 2 years; (**d**) *t* = 3 years; (**e**) *t* = 6 years; (**f**) *t* = 8 years.

Figure 12 and Figure 13 show the simulation results (average value *μ*) of chloride ion concentration in cross-sections of two concrete groups under various corrosion times. It is observed that chloride ions gradually diffuse inward from the concrete surface with increasing corrosion time. The corner of the concrete section exhibits different diffusion characteristics, i.e., the chloride ion diffusion rate at this position is higher than that at the ordinary position, indicating that engineering designers can intuitively understand the most severely corroded parts of the concrete structure in corrosive marine environments through the CA model, which is helpful for engineering designers in durability design decision making of new concrete structures or the maintenance and reinforcement of existing structures.

### 4.2. Chloride Ion Diffusion in Stressed Concrete

**Figure 14:**
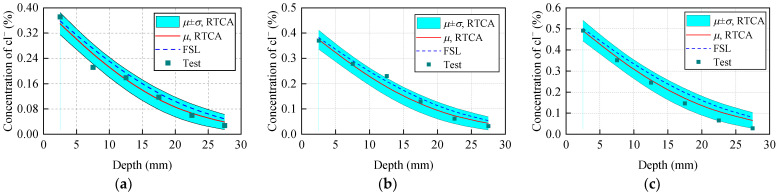
Comparison between the statistical results of the 3D RTCA model and the experimental data and Fick’s second law analytical solution: (**a**) *w*/*c* = 0.35, (**b**) *w*/*c* = 0.4, (**c**) *w*/*c* = 0.45.

The above model accuracy verification using experimental data fails to consider the effect of cross-sectional stress on chloride ion diffusion. Therefore, to verify the effectiveness and accuracy of the proposed model in the analysis of chloride ion erosion in stressed concretes, simulation results are compared with the experimental results in conference [38]. With reference to a sample of 1000 simulations, the comparison between the statistical results (mean value *μ*, standard deviation *σ*) of the 3D RTCA model and the test value is shown in Figure 14. At the same time, in order to facilitate comparison with other theoretical results, Figure 14 also shows the concentration of chloride ions in a cross-section of concrete predicted by FSL. The red solid line represents the statistical results of the model (mean value *μ*), the blue dotted line represents the calculation result of FSL, and the square represents the experimental data.

As shown in Figure 14, except for individual data points, all data are within the interval (− σ: *μ* + σ) predicted by the 3D RTCA model, which shows that the statistical parameters employed by the model can reflect the randomness of chloride ions in the diffusion process. The deviation between the chloride ion concentration of a concrete section predicted by FSL and the test value is greater than that between the model simulation result and the test value. The simulation results are closer to experimental findings than FSL analytical solutions. This is mainly because the FSL analytical solution considers the coefficient Ks as a constant (*K_s_* = 1.0824) during the solution, that is, only the influence of the tensile stress of the lowest edge of the concrete section on the diffusion coefficient is considered, and the change in the stress in the cross-section of concrete with the height of the section is not considered. Compared with FSL, the 3D RTCA model can effectively solve the problem that FSL fails to consider the change of the coefficient of influence with cross-section stress through simple local evolution rules. It is indicated that the 3D RTCA model has higher accuracy when analyzing chloride ion diffusion effect in stressed concretes. It can truly reflect the stress level effect on chloride ion diffusion.

**Figure 15:**
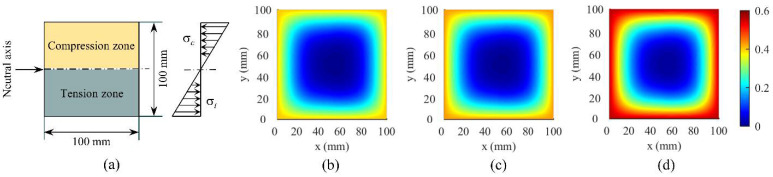
Comparison between the statistical results of the 3D RTCA model, test value, and FSL analytical solution: (**a**) section size and stress distribution; (**b**) *w*/*c* = 0.35; (**c**) *w*/*c* = 0.4; (**d**) *w*/*c* = 0.45.

Figure 15 presents the average simulation results of chloride ion concentration in a stressed concrete cross-section under a variety of water-cement ratios. It is observed that increasing the water–cement ratio increases chloride ion concentration at the same position in the cross-section. The difference in concentration distribution of chloride ions between the concrete compression zone and the tension zone is not obvious, which may be caused by the too-short erosion time.

## 5. Conclusions

In the current research, a cellular automata model to simulate chloride ion 3D distribution in concrete is developed according to cellular automata theory. Using this model, by taking further consideration of the uncertainty and randomness of chloride ion diffusion and the structural stress level effect on the chloride ion diffusion effect, an improved cellular automata model, called the 3D RTCA model, is proposed. The comparison of model prediction statistical results with analytical solutions and experimental data shows that the proposed 3D RTCA model is accurate and can truly reflect the random time-varying characteristics of chloride ion diffusion. The conclusions drawn in this work include the following.

(1) Given the limitations of Fick’s second law analytical solutions in application, this paper introduces a special numerical method for simulating chloride ion diffusion, called cellular automata. Compared with other numerical methods, cellular automata can accurately reproduce linear and nonlinear flow problems with complex boundary conditions without establishing and solving complex partial differential equations. Therefore, it is most appropriate to use cellular automata to simulate chloride ion diffusion in concretes.
(2) A 3D CA model for simulating chloride ion diffusion in concretes is proposed according to chloride ion diffusion characteristics in concrete, which can effectively compensate for the limitations of the application of the 2D cellular automaton model. Compared with the CA model established using the finite element difference method, the 3D CA model developed in the current research, which is based on chloride ion random walk phenomenon in concrete, not only reflects chloride ion diffusion mechanism in concrete, but also has a clearer physical significance. The accuracy of the proposed 3D CA model has been proved by comparison with Fick’s second law analytical solution.
(3) To take into account chloride ion diffusion coefficient variations with corrosion time and stress levels of the cross-section, and the randomness of the diffusion process of chloride ions, a 3D RTCA model has been proposed based on the 3D CA model. By comparing with the results of chloride ion erosion experiments, the accuracy of the 3D RTCA model and the ability to describe the variability of the materials have been proved. Comparison with Fick’s second law analytical solution indicates the effectiveness and superiority of the model in predicting the long-term chloride ion concentration of stressed concrete structures. It can not only describe the time-varying characteristics and randomness of chloride ion diffusion, but also accurately reflect the structural stress level effect on chloride ion diffusion.

## Figures and Tables

**Table 1:** Values of experimental parameters *C_s_*, *D*_0_, and *m*.

Experimental Parameters	Concrete Mix	Mean Value	Coefficient of Variation	Type
*Cs* (%)	PC	0.35	0.1	Normal distribution
	Fly ash	0.5		
*D*_0_ (m^2^/s)	PC	8 × 10^−12^	0.1	Normal distribution
	Fly ash	6 × 10^−12^		
*m*	PC	0.1	—	Deterministic
	Fly ash	0.7		

## Data Availability

The data presented in this study are available on request from the corresponding author.
